# Antibacterial Activities of Herbal Toothpastes Combined with Essential Oils against *Streptococcus mutans*

**DOI:** 10.3390/pathogens8010020

**Published:** 2019-02-01

**Authors:** Özgü İlkcan Karadağlıoğlu, Nuran Ulusoy, Kemal Hüsnü Can Başer, Azmi Hanoğlu, İrem Şık

**Affiliations:** 1 Near East University Faculty of Dentistry, Department of Restorative Dentistry, Near East University, Northern Nicosia, Northern Cyprus, 99138 Mersin 10, Turkey; nuranulusoy@gmail.com; 2 Near East University Faculty of Pharmacy, Department of Pharmacognosy, Near East University, Northern Nicosia, Northern Cyprus, 99138 Mersin 10, Turkey; kemalhusnucan.baser@neu.edu.tr (K.H.C.B.); azmi.hanoglu@neu.edu.tr (A.H.); 3 Inonu University Faculty of Medicine, Department of Microbiology, Inonu University, Elazığ Road, 44280 Malatya, Turkey; iremozdemir89@hotmail.com

**Keywords:** essential oils, *S. mutans*, antibacterial, toothpaste

## Abstract

In recent years, people have become more conscious about the side-effects of fluoride toothpastes and herbal products have drawn attention as alternatives in the struggle against caries. Studies have focused on the benefits of essential oils obtained from herbs because of their antibacterial effects. The aim of this study was to evaluate and compare the antibacterial activity of *Origanum dubium* and *Cinnamomum cassia* oils combined with herbal toothpastes against *Streptococcus mutans*. The antibacterial activity of the test materials was determined using the agar well diffusion method before and after the addition of essential oils. We tested the efficacy of Splat Organic and Splat Biocalcium against *S. mutans* (12 mm and 11 mm, respectively) doubled in combination with *Origanum dubium* (23 mm for both toothpastes) and tripled with *Cinnamomum cassia* (38 mm and 36 mm, respectively). Jack N’ Jill toothpaste, which did not initially show any antibacterial effect, exhibited the largest inhibition zones after the addition of the essential oils (38 mm for *Origanum dubium* and 39 mm for *Cinnamomum cassia*). The results of this study pointed out that herbal toothpastes exhibit statistically higher antibacterial activity against *Streptococcus mutans* (*p* < 0.05) than their initial forms after the addition of essential oils.

## 1. Introduction

Dental caries, one of the most common health problems in the world, is a chronic disease that destroys tooth tissue and that can adversely affect chewing and aesthetic appearance [1]. The most important aspect of the formation of dental caries is that in the absence of plaque or fermentable carbohydrates, caries does not occur [2]. Many factors, such as cariogenic microflora, fermentable carbohydrates, plaque, and duration, are considered as possible sources for the development of caries [3]. Dental caries occurs as a result of the interaction of these factors and host susceptibility, and the acidogenic bacteria often use sucrose as a substrate [4].

A great majority of the evidence on the epidemiology of dental caries suggests that *Streptococcus mutans* (*S. mutans*) is one of the most effective cariogenic bacteria in the initial formation of caries [4]. According to Ritz [5], this pioneer species, in which streptococci predominates for plaque development, are followed by actinomyces.

The use of alcohols and antibiotics, such as ampicillin, penicillin, erythromycin, and chlorhexidine, in traditional treatment modalities are proven methods to prevent dental caries [6]. If there is an increase in resistance to antibiotics in the biofilm bacteria, progression in these pathologies will occur [7]. Due to this resistance of bacteria to antibiotics and traditional treatment methods, the development of anti-infective agents active against microorganisms is being targeted [8].

Antibacterials are the most common agents used to influence bacterial viability in biofilms [9]. While investigating the nature of tooth decay, Miller stated that antiseptics could be used as an active agent in the prevention of caries [10]. One of the most powerful oral hygiene methods that a person can use is fluoride-containing dentifrices that have high clinical efficacy [11]. However, using chemicals containing fluoride can cause intestinal and oral flora changes and even dental staining, vomiting, and oral cancer [12]. Other antimicrobial agents used in the treatment and prevention of oral diseases, such as chlorhexidine, amine fluorides, cetylpyridinium chloride, and products containing such chemicals, have toxic effects and cause tooth stains. Ethanol, which is commonly used in mouthwashes, may cause oral cancer [13,14,15,16,17]. Many bacteria develop resistance to chemicals, such as the antibiotics and antivirals used in the treatment of diseases caused by microorganisms. For this reason, researchers are continuing their search for alternative products to synthetic chemicals [18]. The phytochemicals isolated from the plants used in traditional medicine seem to be a good alternative [19].

Herbal therapies are the main source of medicine in the rural areas of developing countries [20]. Natural products obtained from medicinal plants are the basis of a large number of active biological components that can lead to the development of new chemicals for medicines. The antibacterial, antiviral, and anti-inflammatory activity of herbal products has found its way into dentistry. Some studies have examined the effects of plant extracts and plant products on specific oral pathogens and other researchers focused on the inhibition of biofilm formation, reducing the microbial adhesion that is primarily responsible for dental plaque formation [21,22]. Plant extracts, essential oils, and phytochemicals have been explored in terms of their ability to prevent or cure bacterial adhesion [23]. Essential oils can be used due to their antibacterial activities against several bacteria [24,25,26], including *S. mutans* [27]. These bactericide or bacteriostatic effects of essential oils can be explained by their components, such as terpenes and terpenoids, which are all characterized by a low molecular weight, and aromatic and aliphatic constituents [28].

There are some studies in the literature comparing fluoride-containing toothpastes and herbal toothpastes [29,30]. Due to their antimicrobial effects, essential oils may be used to prevent dental caries. There are studies in the literature about the effect of essential oil-containing mouthrinses and mouthwashes against different oral bacteria [31,32,33]. There is only one study that examined the effect of the *Thymus vulgaris* essential oil against *Streptococcus mutans*, evaluating the colour, odour, and general appearance of a test toothpaste containing *Thymus vulgaris* [32]. This study aimed to develop a new toothpaste formulation. However, there is no reported study about the anticariogenic effects of *Origanum dubium* (*O. dubium*) and *Cinnamomum cassia* (*C. cassia*) oils combined with herbal toothpastes. Thus, the present study aimed to evaluate the antibacterial activity of various herbal toothpastes against *S. mutans* before and after adding *O. dubium* and *C. cassia* oils obtained from oregano and cinnamon plants.

## 2. Results

Following GC/MS (Gas chromatography/mass spectrometry) analysis, the components of *Origanum dubium* Boiss. (Lamiaceae) (*O. dubium*) and *Cinnamomum cassia* (L.) J. Presl. (Lauraceae) (*C. cassia*) oils and their relative percentage amounts are shown in Table 1 and Table 2. The major components of *O. dubium* and *C. cassia* were identified as *Carvacrol* (88.3%) and *Cinnamaldehyde* (91.8%), respectively.

**Figure 1:**
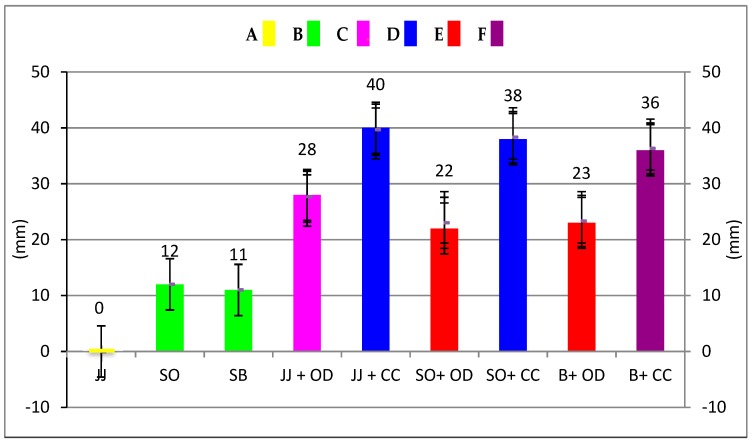
Antibacterial activity of herbal toothpastes before and after essential oil addition. Same capital letters and same coloured columns indicate no statistically significant difference (*p* > 0.05). Different capital letters and different coloured columns indicate statistically significant differences (*p* < 0.05). y-axis shows the inhibition zones of the toothpastes against *S. mutans*. (JJ: Jack N’ Jill; SO: Splat Organic; SB: Splat Biocalcium; JJ + OD: Jack N’ Jill + *O. dubium* essential oil; JJ + CC: Jack N’ Jill + *C. cassia* essential oil; SO + OD: Splat Organic + *O. dubium* essential oil; SO + CC: Splat Organic + *C. cassia* essential oil; B + OD: Biocalcium + *O. dubium* essential oil; B + CC: Biocalcium+ *C. cassia* essential oil).

The data in Table 3 and Figure 1 show the inhibition zone measurements of the test andthe statistical differences. The *C. cassia* essential oil was more effective (38 mm) than the *O. dubium* (30 mm) essential oil. Statistically significant differences were found for the growth inhibition zones of the toothpastes and the toothpaste–essential oil combinations (*p* < 0.05). The highest antibacterial activities were observed with the *C. cassia* essential oil and its combination with Jack N’ Jill and Splat Organic, and there was no statistical difference between them (*p* > 0.05). The *C. cassia* combination with Splat Biocalcium showed lower antibacterial activity than the other *C. cassia* groups (*p*<0.05), but higher antibacterial activity than all the other groups (*p* < 0.05). The lowest antibacterial activities were observed for distilled water, which was the negative control group of this study, and Jack N’ Jill, with no statistical difference between them (*p* > 0.05). Regarding the toothpastes and control groups, *C. cassia* showed higher antibacterial activity than *O. dubium* (*p* < 0.05). *O. dubium* in combination with Jack N’ Jill showed higher antibacterial activity than the combination with Splat Organic and Splat Biocalcium (*p* < 0.05), where there was no statistical difference between these two groups (*p* > 0.05). After the addition of essential oils, the antibacterial activity of the toothpastes was observed to be higher than Colgate Total, which was the positive control group of this study (*p* < 0.05). In addition, there was no statistical difference between Colgate Total, Splat Organic, and Splat Biocalcium (*p* > 0.05).

According to the results of the “test of between subjects effect” (Table 4), all of the factors (types of toothpaste and types of essential oils) were included in the analysis. The interactions between these factors had a significant effect on the results of this study (*p* < 0.001).

## 3. Discussion

Dental caries is a multifactorial disease with high counts of cariogenic bacteria. *S. mutans* is the most frequently isolated bacteria from human dental plaque and was used in the present study because it is believed to be the major cariogenic microorganism of dental caries. The destruction of superficial tooth structures is caused by acids of *S. mutans* and the by-products of carbohydrate metabolism [23]. The reason for the cariogenic potential of *S. mutans* is due to its virulence factors, mainly adhesion capacity, acidogenicity, and aciduricity.

Due to the cariogenic potential of *S. mutans*, oral hygiene measures are used to reduce its accumulation on oral biofilm. Professional care with tooth brushing methods is capable of reducing caries, gum inflammation, and periodontal disease. When looking at the increase in the prevalence of such oral diseases worldwide, it can be stated that only brushing is not enough to prevent tooth decay. For this reason, chemotherapeutic adjuncts may be added to the routine brushing process [34]. Most of the toothpastes recommended by the WHO (World Health Organization), ADA (American Dental Association), and FDI (World Dental Federation) contain fluoride and triclosan [34]. The use of triclosan in toothpastes has been shown to reduce in vivo bacterial viability and gingiva and plaque index scores [35]. Although the use of fluoride and triclosan-containing toothpastes has benefits, the excess use of these chemical and synthetic products also has negative effects. On this basis, it is wise to prefer herbal toothpastes rather than toothpastes containing harsh chemicals. It has been reported that herbal toothpastes and plant extracts exhibited significant results to cure various diseases, like gingivitis, gum bleeding, bad breath and dental caries, besides their anti-cancerous properties. However, there are no studies comparing herbal toothpastes with added essential oils and fluoride toothpastes. The present study aimed to evaluate and compare the inhibitory efficacy of three herbal toothpastes against the cariogenic bacteria, *S. mutans*, before and after adding two essential oils. Colgate Total was used as the positive control group in this study, because it is considered as the gold standard for combating dental caries.

Essential oils for use in the cosmetics and food industry are derived from cinnamon bark, leaves, flowers, and fruit. In traditional Chinese medical treatments, cinnamon has also been used in the treatment of diabetes [36], inflammation, urinary tract infections, and gastrointestinal disorders [37,38].

There are several methods for obtaining essential oils. One of them is the hydrodistillation (HD) method, which is a kind of steam distillation, developed by French Pharmacopoeia, and used in the extraction and quality control of essential oils from dried plants [39]. This method is the oldest method, but it is easier than the other methods [40]. Isotropic distillation forms the basis of extraction. The distillation time may vary depending on the plant to be extracted. Although the distillation time is long, a small amount of volatile oil is obtained. However, undesirable compound and oxidation products can be separated from the essential oils [39]. Because of these advantages, the HD method was preferred for obtaining the essential oils in this study.

Essential oils contain several diverse chemicals and each of these compounds may exhibit different antibacterial activities due to the variations in their mode of action [41]. Gas chromatography/mass spectrometry (GC/MS) analysis is necessary for the determination of the composition of essential oils [42]. The characterization of the essential oil components used in this study was carried out through a comparison of their relative retention times with those of authentic samples or by comparison of their relative retention index (RRI) to a series of n-alkanes. Computer matching against commercial (Wiley GC/MS Library, MassFinder 3 Library) [43,44] and in-house libraries, “Başer Library of Essential Oil Constituents”,of the genuine compounds and components of known oils, as well as MS (mass spectral literature data [45,46], was used for the characterization of the components.

Oregano is a term that describes several species that contain carvacrol as a main component in their structure [47]. Phytochemical and biological studies of the *Origanum* species have proved that it is a rich source of compounds with insecticidal, antibacterial, antifungal, antioxidant, and anti-carcinogenic activities [48,49]. There have been a number of studies on the antibacterial activity of the *Origanum* species. Previous studies investigated the antibacterial activity of *Origanum* species included *O. dubium* on different bacterial strains, such as *E. coli*, *S. aureus*, *C. albicans* [37,50], and *S. mutans* [51,52]. *Origanum* species are defined according to the types of phenolic compounds they contain [53,54]. The main components of oregano oil are carvacrol and thymol. They are monoterpenic phenols and are byosynthesized from γ-terpinene [55] through *p*-cymene [56]. Sivripolou et al. showed in their study that the essential oils obtained from the *Origanum* species have antibacterial properties because of their phenolic compounds, such as carvacrol, thymol, *p*-cymene, and γ-terpinene [57].

The results of this study are consistent with previous research that reported that the antimicrobial and antioxidant activity of *O. dubium* is related to its high carvacrol content [50]. According to the GC/MS analysis in this study, carvacrol was found to be the major component (88.3%) of *Origanum dubium* oil. Therefore, the antibacterial effect of oregano oil in the present study may be related to the carvacrol that it contains. Previous studies on the antibacterial mechanisms of action of plant essential oils including the *O. dubium* essential oil have shown that hydrophobic bioactive compounds may cause cell damage, increase cell membrane permeability, affect ATP production and protein synthesis, cause cellular pH deterioration, and cause cytoplasmic changes [58,59,60]. Hence, its use is limited due to its highly volatile character and undesirable organoleptic properties [61,62]. Therefore, in this study, the essential oils were not used in their pure form.In scientific studies, essential oils are diluted in ethanol, tween, or DMSO (dimethylsulfoxide) [63,64]. Ethanol has been shown to alter the activity of the material due to its antibacterial activity [65]. Therefore, in this study, DMSO was preferred to reduce side effects and allow the oils to dissolve homogeneously in the toothpaste. The safety of the formulations was evaluated through the determination of the DMSO additions that would not affect the antibacterial activity of the materials in the literature [66].

The genus, *Cinnamomum*, falls within the family, Lauraceae, and contains more than 300 evergreen aromatic trees and briars [67]. *Cinnamomum cassia* (known as Chinese cinnamon) [68] was purchased from local market and used in this study. There have been studies on the antibacterial activity of cinnamon [29,65,69]. Furthermore, there havealso been studies related tothe strongantibacterial properties of cinnamaldehyde [70,71]. Using GC/MS analysis, cinnamaldehydewas found to bethe major component of *C. cassia* (91.79%). The amount of cinnamaldehydefor different species of essential oils varied (50% to 88%) in the literature [72,73]. The amount of cinnamaldehyde used in this study was compatible with of Ooi et al. and Singh et al. [72,74].

The pure essential oils used in the present study were applied to agar plates to be sure that they were not contaminated before adding them to the tested toothpastes. It was observed that the diameter of the inhibition zones of the pure oils were very large. This can be attributed to the fact that pure essential oils cause oxidative stress. However, it was very difficult to distinguish the haemolysis zone and the inhibition zone on the agar plates, especially for the *O. dubium* oil.

To determine the antibacterial activity of pure essential oils, the agar-disc diffusion method was preferred in this study. The agar-disc diffusion method is a common method for evaluating the antimicrobial activities of plants or their extracts [75]. There are several methods to determine their antibacterial activity in the literature, such as the minimum inhibitory concentration determination method, two variants of the bioautographic method—the direct variant of the bioautographic method (chromatogram layer) and the indirect variant of bioautographic method (agar diffusion)—and two variants of the agar diffusion method (well and disc) [76]. As essential oils are highly viscous and have a hydrophobic structure, testing the antimicrobial activity of essential oils is not easy. The other methods have some disadvantages, as they cannot be mixed homogenously and leakage of essential oils may occur from the well into the agar. For these reasons, the agar-disc diffusion method was used in this study.

The comparison of the pure essential oils demonstrated that *C. cassia* oil had a larger inhibition zone (38 mm) than *O. dubium* oil (30 mm). The inhibition zone of the pure essential oil of *C. cassia* against clinically isolated human *S. mutans* was measured as 19 mm in a previous study by Chaudhry and Tariq [77]. This variation with our results may be due to the differences in the active ingredient ratios of the essential oils.

The agar-disc diffusion method was preferred to prevent the volatilization and dispersion of oils in the evaluation of essential oils, but this method was not suitable for the evaluation of the toothpaste–oil mixtures. Thus, the agar-well diffusion method was chosen to determine the antibacterial activity of the toothpastes and the toothpaste–oil mixtures. The implementation of the agar-disc diffusion method is also limited in some countries because the discs are very expensive. Magaldi [78] developed a similar method named the“agar-well diffusion method”, in which test materials are placed in wells opened into the agar. Several researchers used this method to evaluate the antibacterial effects of some materials [63,79]. Magaldi’s [78] well diffusion method was also preferred in this study to ensure that the oil-containing toothpastes were homogeneously absorbed by the disc.

Splat Organic, without the addition of essential oil, had a similar effect to Colgate Total. Splat Organic contains *Aloe barbadensis* Mill. (*Aloe vera* Mill) gel and papain and *Melaleuca alternifolia* (Maiden et Betche) Cheel *(M. alternifolia)* leaf extract. *Aloe vera* is a well-known medicinal plant of the Liliaceae family. The pharmacological actions of *Aloe vera* gel, including anti-inflammatory, antibacterial, antioxidant, immune-boosting, and hypoglycaemic properties, have been studied in vitro and in vivo [80,81,82,83,84]. An in vitro study showed that an *Aloe vera* gel was as effective as commercially popular dentifrices in controlling *S. mutans*, *C. albicans*, *S. sanguis*, and *A. Viscosus* growth. The *Aloe vera* gel showed an antibacterial effect, especially against *S. mitis* [85] and *S. mutans* [86]. In a randomized controlled clinical trial for 6 months, toothpaste containing an aloe vera gel was used, and measurements were recorded at 6 weeks, 12 weeks, and 24 weeks. During this time, the gingival and plaque index scores decreased and there was a significant improvement in the microbiological counts [87]. Papain is a proteolytic enzyme obtained from the papaya (*Carica papaya* L.) plant and has the strongest effect among all papaya products [88,89]. Papain, with its proteolytic activity against the amino acid, has been extensively used in the fields of medicine and food [90]. Papain-containing materials have shown successful results when used against *S. mutans* biofilm [89,91]. *M. alternifolia* is known as “tea tree”. The antibacterial activity of this plant has been utilized for many years. Several studies found that the *M. alternifolia* essential oil and its main components are effective against pathogenic flora. Due to its broad spectrum, a strong inhibition against gram-positive and gram-negative bacteria, including *A. actinomycetemcomitans*, *S. gordonii*, and *S. mutans*, was found [92,93]. The antibacterial effect of Splat Organic may be due to its *Aloe vera* and papain content. Splat Biocalcium does not contain fluoride, triclosan, chlorhexidine, and alcohol, but contains nano-hydroxyapatite and papain like Splat Organic. The similar antibacterial effect of Splat Biocalcium to the positive control group may be due to its papain content.

Jack N’ Jill herbal toothpaste does not contain any preservatives and flavours because it is produced for infants, toddlers, and children. According to the producer, it contains *Calendula officinalis* L. and, in the literature, the antimicrobial activity of this plant was shown in a herbal mouthrinse that contains ethanol [94]. The antibacterial activity of the mouthrinse may also be explained by the fact that it contains ethanol, a solvent that has antibacterial effects. However, in the present investigation, as Jack N’ Jill toothpaste does not contain any solvent, like ethanol, in its content, it did not show any antibacterial activity against *S. mutans* before the addition of essential oils. Jack N’ Jill toothpaste contains xylitol. Xylitol is a naturally occurring sugar alcohol in most plants and has been approved for use as a sweetener in the food industry by the US Food and Drug Administration since 1963. Xylitol cannot be fermented by oral microorganisms and has been shown to reduce *S. mutans* levels in plaque and saliva and markedly reduce tooth decay. The antibacterial effect of xylitol have been evaluated by various studies [95,96], but the amount added to the materials may change the efficacy. According to a study, it was found that it did not show any antibacterial effect under a certain level [97]. The reason that Jack N’ Jill toothpaste did not exhibit any antibacterial activity in the present study may be due to its low xylitol content.

**Figure 2:**
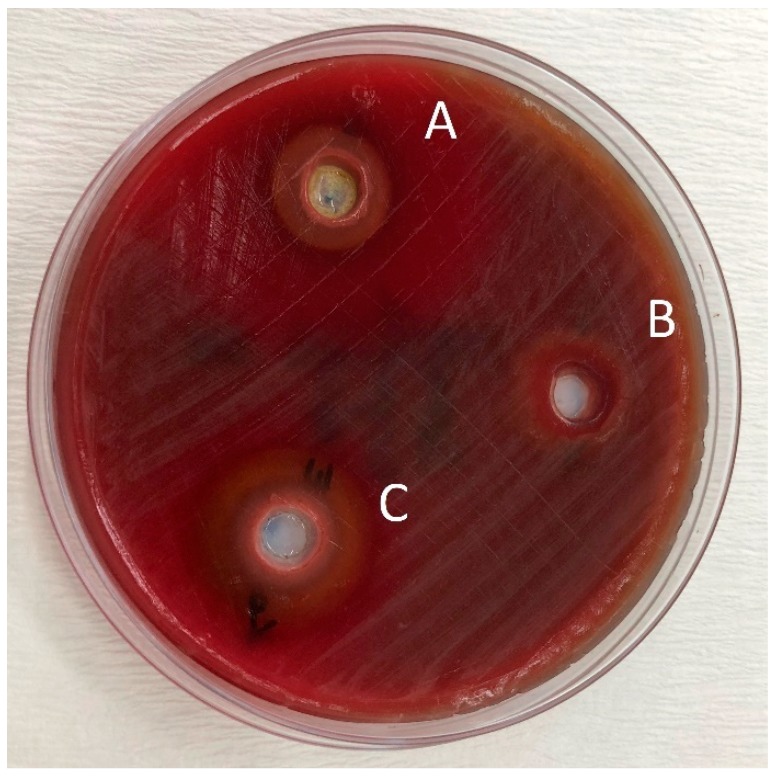
Zones of inhibition produced by the Splat Organic in combination with essential oils and the control group in the agar-well diffusion test. (**A**): toothpaste—*O. dubium* oil combination; (**B**): toothpaste—distilled water combination; (**C**): toothpaste—*C. cassia* oil combination.

**Figure 3:**
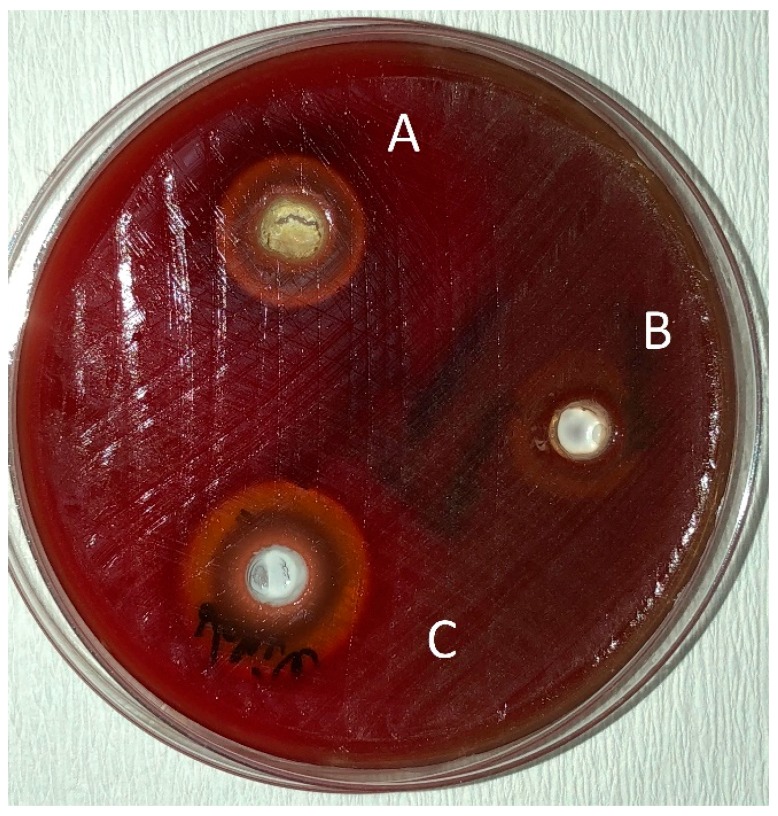
Zones of inhibition produced by Splat Biocalcium in combination with essential oils and the control group in the agar-well diffusion test. (**A**): toothpaste—*O*. *dubium* oil combination; (**B**): toothpaste—distilled water combination; (**C**): toothpaste—*C*. *cassia* oil combination.

**Figure 4:**
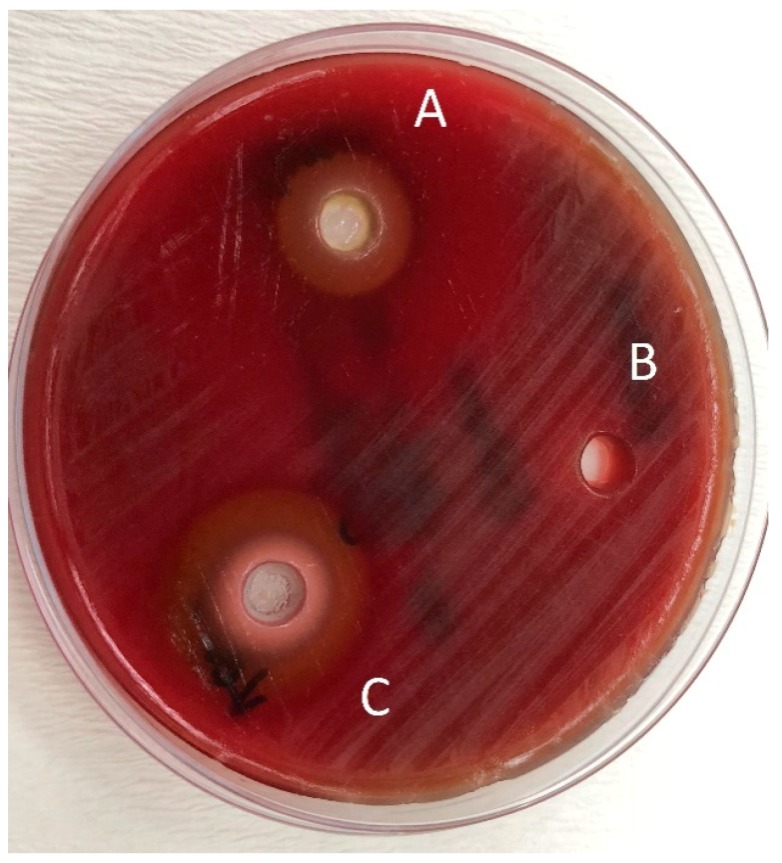
Zones of inhibition produced by the Jack N’ Jill in combination with essential oils and the control group in the agar-well diffusion test. (**A**): toothpaste—*O. dubium* oil combination; (**B**): toothpaste—distilled water combination; (**C**): toothpaste—*C. cassia* oil combination.

Bhattacharjee evaluated the antibacterial activity of toothpastes with different properties and reported that herbal toothpastes are more effective than fluoride- and triclosan-containing toothpastes [30]. According to the comparison of antibacterial activity of the toothpastes tested, Splat Organic and Splat Biocalcium without essential oil addition showed similar diameter inhibition zones to Colgate Total against *S. mutans* (*p* < 0.05) (Table 3). This result shows that fluoride is not the only ingredient that causes antimicrobial activity in toothpastes. In this study, particularly oregano oil extracted from *O. dubium*, a native plant from the Yeşilırmak region in Cyprus, and the oil extracted from *C. cassia* were added to the herbal toothpastes to investigate their antimicrobial potential against *S. mutans.* Although Splat Organic and Splat Biocalcium without any essential oil addition showed some antibacterial activity against *S. mutans*, their efficacy doubled in combination with *O. dubium* oil and tripled with *C. cassia* oil (Table 3) (Figure 2 and Figure 3). Jack N’ Jill toothpaste, which showed no antimicrobial activity against *S. mutans*, surprisingly gained statistically significant efficacy in combination with *O. dubium* oil and *C. cassia* oil (*p* < 0.05) (Table 3 and Figure 4). This result may be due to the synergistic interaction of calendula in the content of the toothpaste, with the high concentration of cinnamaldehyde in the added *C. cassia* oil. According to the results obtained from the “test of between subject effect”—which tested the interactions between the toothpastes, essential oils, and toothpaste–essential oil combinations—there were statistically significant differences among all groups (*p* < 0.001, R^2^ ≥ 80). Although the type of essential oil and the interactions between the toothpastes and the essential oils had higher effects on the results (99% and 98%, respectively), the type of toothpaste had a lesser effect on the results (77%).

The herbal toothpastes with added *C. cassia* oil exhibited larger inhibition zones against *S. mutans* compared to the herbal toothpastes with the combination of oregano oil. The inhibition zones of Jack N’ Jill toothpaste and Splat Organic were similar in combination with *C. cassia* oil. However, the Splat Biocalcium and *C. Cassia* oil combination was found to be less effective against *S. mutans* compared to these two herbal toothpastes. The data obtained cannot be compared with other studies because there is no study on the antibacterial activities of the tested toothpastes and essential oils in the literature. These findings may not correspond to the actual toothpaste behaviour and clinical potential. An experiment with extracted teeth will provide additional data to support the validity of the toothpaste–essential oil combinations in in vitro conditions. Despite the importance of in vitro studies, long-term, clinical trials show the best scientific results regarding treatments and the true response of treatments should be determined with independent clinical trials.

Within the limitations of this study, the efficacy of Splat Organic and Splat Biocalcium against *S. mutans* doubled with *O. dubium* and tripled with *C. cassia* combination. In addition, Jack N’ Jill gained antibacterial activity and showed the best effect against *S. mutans*. Therefore, it can be concluded that there was a statistically significant increase in the antibacterial activity of herbal toothpastes with the addition of essential oils (*p* < 0.05) and the effect of *C. cassia* oil was significantly higher than that of *O. dubium* oil (*p* < 0.05). Alongside these promising findings, further investigations into toothpastes with added essential oil may lead to improvements in the formulation of toothpastes to optimize their anti-caries activity.

## 4. Materials and Methods

Three herbal toothpastes, a fluoride-containing toothpaste (positive control group), and distilled water (negative control group) were used. Table 5 shows the ingredients of the tested toothpastes.

### 4.1. Obtaining Essential Oils

The herbal partsof *O. dubium* were collected from Yeşilırmak, Turkish Republic of Northern Cyprus in season and air dried. *O. dubium* oil was obtained from the leaves using a Clevenger apparatus (Ildam, Ankara, Turkey) for 3 h using the water distillation method [34]. The *C. cassia* bark (Senfoni, Nicosia, TRNC), purchased from a local market, were subjected to the same procedure as *O. dubium*. The organic layer of each essential oil was separated, dried over anhydrous sodium sulphate (Na_2_SO_4_), and filtered with a 0.45 µm sterile syringe filter unit (Miller-HV, Merck KGaA, Darmstadt, Germany). The oils were dissolved in %10 dimethyl sulphoxide (DMSO) (VWR Chemical, Paris, France) for use in the toothpaste. The oils were kept at +4 °C until use.

### 4.2. GC/MS Analysis of Essential Oils

#### 4.2.1. GC/MS Analysis

The GC-MS analysis was carried out with an Agilent 5975 GC-MSD system. An Innowax FSC column (60 m × 0.25 mm, 0.25 mm film thickness) was used with helium as the carrier gas (0.8 mL/min). The GC oven temperature was kept at 60 °C for 10 min and programmed to 220 °C at a rate of 4 °C/min, and kept constant at 220 °C for 10 min and then programmed to 240 °C at a rate of 1 °C/min. The split ratio was adjusted at 40:1. The injector temperature was set at 250 °C. Mass spectra were recorded at 70 eV. The mass range was from *m*/*z* 35 to 450 [42].

#### 4.2.2. GC Analysis

The GC analysis was carried out using an Agilent 6890 N GC system. The FID (Flame Ionization Detector) detector temperature was 300 °C. To obtain the same elution order with GC-MS, simultaneous auto-injection was carried out on a duplicate of the same column applying the same operational conditions. The relative percentage amounts of the separated compounds were calculated from FID chromatograms. The analysis results are provided in Table 1 and Table 2.

##### Determination of the Antibacterial Effect of Oils

In this part of the study, the disc diffusion method was used in accordance with the EUCAST (European Committee on Antimicrobial Susceptibility Testing) recommendations [70,75]. Sterile filter paper disks, 6 mm in diameter, were impregnated with 45 µL of each essential oil directly and discs was left to dry for 15 min. The plates were inoculated with *S. mutans* ATCC 35668 (ATCC, Manassas, VA, USA) to examine the antibacterial activity. Bacteria were cultured on Columbia agar with 5% sheep blood (BioMerieux SA, Marcy-l’Etolle, France) and were grown anaerobically at 37 °C for 24 h (EC 160 CO_2_ Incubator, Nuve, Ankara, Turkey). Several colonies of cultured bacteria were transferred in inoculum saline (MicroScan Inoculum Saline, Beckman Coulter, Inc., Brea, CA, USA) and the density was adjusted to McFarland Standard 0.5 (MicroScan Turbidity Meter, Siemens, Deerfield, IL, USA). A total of 0.1 mL bacterial suspension in inoculum saline taken by sterile ecuvion was spread to the Mueller-Hinton Agar plates (90 mm in diameter) with 5% defibrinated horse blood and 20 mg/L β-NAD (β-nicotinamide adenine dinucleotide) (BioMerieux SA, Marcy-l’Etolle, France). Then, the paper discs impregnated with essential oils were placed on inoculated agar plates and were grown at 37 °C for 24 h (EC 160 CO_2_ Incubator, Nuve, Ankara, Turkey). Antibacterial activity was evaluated by measuring the diameter of inhibition zone (DIZ) of the tested bacteria.

### 4.3. Determination of the Antibacterial Effect of Toothpastes

The antibacterial effects of toothpastes were determined using the agar-well diffusion method. Mueller-Hinton Agar plates (90 mm in diameter) with 5% defibrinated horse blood and 20 mg/L β-NAD (BioMerieux SA, Marcy-l’Etolle, France) were used. Up to three wells were created in the agar of each plate by removing plugs cut with a sterile 6 mm diameter stainless steel biopsy punch. 45 milligrams of each toothpaste used in this study was diluted with 45 µL distilled water and homogenized in a vortex mixer (Combi-Spin, BioSan, Riga, Latvia) for 3 min. The sonicator (Gen-probe, San Diego, CA, USA) was applied for 5 min in the finalizer to complete the dissolution. Distilled water was used as a control group.

The materials were loaded into the wells opened on the agar plates. After loading the wells, the plates were inoculated with *S. mutans* ATCC 35668 (ATCC, Manassas, VA, USA). Then, the same procedure as described in the section “determination of the antibacterial effects of oils” was applied.

The diameters of each area of growth inhibition were measured to the nearest 0.5 mm by viewing the bottom of the agar plate. Three replicates were performed for each agent. Antibacterial activity was evaluated by measuring the diameter of the inhibition zone (DIZ) of the tested bacteria.

### 4.4. Mixing the Herbal Toothpastes with Essential Oils and Determination of the Antibacterial Effect of Toothpastes Combined with Essential Oils

A total of 45 milligrams of each commercial herbal toothpaste (Jack N’ Jill Flavour Free Natural Toothpaste, Melbourne, Australia; Splat Organic (Splat, Russia), Splat Biocalcium (Splat, Russia) were mixed homogeneously with 45 µL of each diluted essential oil. After the addition of 100 µL of distilled water, the toothpaste–oil mixtures were homogenized in a vortex mixer (Combi-Spin, BioSan, Riga, Latvia) for 3 min. The sonicator (Gen-probe, San Diego, CA, USA) was applied for 5 min in the finalizer to complete the dissolution. A fluoride—containing toothpaste (Colgate Total, Colgate-Palmolive Co., New York, NY, USA) was used as positive control group, whereas distilled water was used as a negative control group.

The antibacterial effects of the experimental and control groups were determined using the agar-well diffusion method. For this purpose, Mueller-Hinton Agar plates 90 mm in diameter with 5% defibrinated horse blood and 20 mg/L β-NAD (BioMerieux SA, Marcy-l’Etolle, France) were used. Up to three wells were created in the agar of each plate by removing plugs cut with a sterile 6 mm diameter stainless steel biopsy punch. Each plate contained one well of toothpaste–oregano oil mixture, 100 µL of toothpaste–cinnamon oil mixture, and 100 µL of toothpaste–distilled water mixture for the control. A total of 100 µL fluoride toothpaste and 100 µL of distilled water were also tested with three replicates on two different plates. The process was carried out in triplicate for the herbal toothpastes with oregano oil or cinnamon oil.

The same procedure as used for the determination of the antibacterial effect of the toothpastes was applied to grow the bacteria and spread them to the agar plates containing the toothpastes and measure the diameter of the inhibition zones. The diameters of each area of growth inhibition were measured to the nearest 0.5 mm by viewing the bottom of the agar plate. A total of 15 replicates were performed for each agent. The antibacterial activity was evaluated by measuring the diameter of the inhibition zone (DIZ) of the tested bacteria.

### 4.5. Statistical Analysis

Descriptive statistics were performed for each group and the distributions of the toothpastes with and without essential oils were checked by normality tests (Shapiro–Wilk). Three-way ANOVA was performed to determine the statistical difference among all groups according to the inclusion of essential oils and different testing methods of the antibacterial activities. A separated three-way ANOVA was performed to analyse the effect of the toothpastes, essential oils, and the interaction of the toothpaste–essential oils by ignoring the control groups. The Tukey test was applied for pairwise comparison with 95% confidence intervals, where *p* < 0.05 (Bonferroni adjusted alpha = 0.05) was accepted as statistically significant.

Tests of between-subject effects were performed to find out which factors and interactions (partial eta squared values of material type and essential oil type) were affecting the results and to find out the effect size (R^2^) of the ANOVA tests. A result of *p* < 0.001 indicated that the effect on the result of that parameter was high. R^2^ ≥ 80 indicated that the total effect of the parameters included in the measurement was about 80% and the reliability of the statistical analysis and the results were too high.

## Figures and Tables

**Table 1:** Essential oil composition of *O. dubium*.

RRI	Compound Name	Relative Percentage Amounts (%) A%
1020	α-Pinene	0.3
1024	α-Thujene	0.5
1172	Myrcene	0.4
1177	α-Phellandrene	0.2
1192	α-Terpinene	0.9
1211	Limonene	0.1
1223	β-Phellandrene	0.1
1260	γ-Terpinene	2.7
1288	*p*-Cymene	3.8
1299	Terpinolene	0.1
1478	trans-sabinene hydrate	0.4
1556	Linalool	0.1
1565	cis-sabinene hydrate	0.1
1625	Terpinene-4-ol	0.7
1629	β-Caryophyllene	0.1
1639	trans-dihydrocarvone	Tr
1718	α-Terpineol	0.5
1728	Borneol	0.1
1771	Carvone	Tr
2108	Elemol	0.1
2159	Spathunelol	0.1
2210	Thymol	0.2
2243	Carvacrol	88.3
2273	β-Eudesmol	0.1
	**Total**	100.0

Table showing the compounds of *O. dubium* oil. A: *O. dubium* essential oil, %: calculated from FID (Flame Ionization Detector) data, RRI: Relative retention indices calculated against n-alkanes, Tr: Trace (<0.1).

**Table 2:** Essential oil composition of *C. cassia* essential oil.

LRI	Compound	Relative Percentage Amounts (%) B%
1021	α-pinene	0.61
1073	Camphene	0.67
1119	β-pinene	0.17
1212	Limonene	0.23
1221	1,8-cineole	1.18
1288	*p*-cymene	0.08
1515	α-cubebene	0.07
1556	Benzaldehyde	0.46
1605	Bornyl acetate	0.54
1625	Terpinen-4-ol	0.48
1629	β-caryophyllene	0.05
1718	α-terpineol	0.83
1728	Borneol	0.19
1787	δ-cadinene	0.03
1818	Benzenepropanal (=phenylpropyl aldehyde)	0.34
1940	(*Z*)-cinnamaldehyde	0.06
2091	(*E*)-cinnamaldehyde	91.79
2104	1-epi-cubenol	1.16
2188	Cinnamyl acetate	0.72
2242	Carvacrol	0.01
2514	Coumarin	0.19
**Total**		99.84

Table showing the compounds of *C. cassia* essential oils. B: *C. cassia* essential oil, %: calculated from FID (Flame Ionization Detector) data.

**Table 3:** Mean ± standard deviation, minimum and maximum values of zones of inhibition against *S. mutans* with essential oils, herbal toothpastes, and control groups.

Groups		Min	Max	Mean ± SD
***O. dubium* Oil**	-	30	31	30.33 ± 0.58 ^a^
***C. cassia* Oil**	-	38	40	38.67 ± 1.15 ^b^
**Colgate Total**	-	12	12	12.00 ± 0.00 ^c^
**Jack N’ Jill**	-	0	0	0.00 ± 0.00 ^d^
	*+O. dubium* Oil	27	28	27.67 ± 0.58 ^c^
	*+C. cassia* Oil	39	40	39.67 ± 0.58 ^b^
**Splat Organic**	-	12	12	12.00 ± 0.00 ^c^
	*+O. dubium* Oil	22	24	23.00 ± 1.00 ^f^
	+*C. cassia* Oil	38	39	38.33 ± 0.58 ^b^
**Splat Biocalcium**	-	11	11	11.00 ± 0.00 ^c^
	+*O. dubium* Oil	23	24	23.33 ± 0.58 ^f^
	+*C. cassia* Oil	36	37	36.33 ± 0.58 ^g^
**Distilled Water**	-	0	0	0.00 ± 0.00 ^d^

The same lower-case letters indicate no statistically significant difference (*p* > 0.05). Different lower-case letters in columns indicate statistically significant differences (*p* < 0.05).

**Table 4:** “Test of between subject effect” showing the interactions between the toothpastes, essential oils, and toothpaste and essential oil combinations.

	TP	EO	TP + EO
Inhibition Zone	0.000	0.000	0.000
Partial eta Squared	0.772	0.999	0.983

R^2^: 0.999. Table showing interactions between tested materials. *p* < 0.05 means that the parameter has an effect on the results. *p* < 0.001 means the effect of that parameter on the results is high. TP: Toothpaste, EO: Essential oils, TP + EO: Toothpastes + Essential Oils. R^2^: effect size, percentage of total effect of the parameters included in the study.

**Table 5:** Toothpaste ingredients.

Toothpaste	Ingredients
Colgate Total	Sodium Fluoride (0.24% (0.14% *w*/*v* Fluoride Ion)), Triclosan (0.30%), Water Hydrated Silica, Glycerine, Sorbitol, PVM/MA Copolymer, Sodium Lauryl Sulphate
Splat Organic	Cellulose Gum, Flavour, Sodium Hydroxide, Carrageenan, Propylene Glycol, Sodium Saccharin, Titanium Dioxide Hydrogenated Starch Hydrolysate, Aqua, Hydrated Silica, PEG-8, Sodium Lauryl Sulphate, Aroma, Glycerine, Calcium Lactate, *Aloe barbadensis* Leaf Extract, Xanthan Gum, *Melaleuca alternifolia* Leaf Oil, Sodium Methylparaben, o-Cymen-5-ol, Papain, Citric Acid, Sodium Benzoate, CI 19140, CI 42090, Limonene.
Splat Biocalcium	Calcium Lactate, Sodium Bicarbonate, Hydroxyapatite, PVP, Fish Oil, Papain.
Jack N’ Jill	Xylitol, Purified water, Gylicerin, Silica, Xanthan gum, Organic *Calendula officinalis* extract, Potassium sorbate, Citric acid

Table showing the ingredients of the toothpastes. All of the toothpastes were herbal based except Colgate Total.
